# Evaluating osteointegration of implants with low insertion torque in soft bone

**DOI:** 10.6026/973206300213094

**Published:** 2025-09-30

**Authors:** Nazia Afreen, Ankita Barodiya, Ramjee Lal Raigar, Ayushi Gupta, Satish Makwana, Mugur Basavanna Jayaprakash, Miral Mehta, Jugajyoti Pathi

**Affiliations:** 1 Department of Prosthodontics, AI Badar Dental College and Hospital, Kalaburagi, Gulbarga, Karnataka, India; 2 Department of Dental Surgery, Government Medical College, Seoni, Madhya Pradesh, India; 3 Department of Prosthodontics, Government Dental College, Jaipur, Rajasthan, India; 4 Department of Prosthodontics, Triveni Institute of Dental Sciences, Hospital & Research Centre, Bilaspur, Chhattisgarh, India; 5 Dental Department, CU Shah Medical College and Hospital, Surendranagar, Gujarat, India; 6 Department of Prosthodontics, Dental Institute(Govt), RIMS, Ranchi, Jharkhand, India; 7 Department of Pediatric and Preventive Dentistry, Karnavati School of Dentistry, Karnavati University, Gandhinagar, Gujarat, India; 8 Department of Oral & Maxillofacial Surgery, Kalinga Institute of Dental Sciences, KIIT Deemed to be University, Bhubaneswar, Odisha, India

**Keywords:** Dental implants, insertion torque, osseointegration, soft bone, primary stability, animal model

## Abstract

Achieving primary stability in low-density bone is a major challenge in implant dentistry, with high insertion torque (HIT) causing bone micro damage. Therefore, it is of interest to evaluate osteointegration of implants placed with low insertion torque (LIT, 15-25 Ncm) versus HIT (35-45 Ncm) in sheep femoral condyles. At 12 weeks, bone-to-implant contact (58.3% vs. 62.1%, p=0.18) and bone volume fraction (35.4% vs. 38.2%, p=0.22) were comparable between groups. Initial implant stability was lower with LIT but equalized with HIT at 12 weeks. Thus, LIT implants achieved similar osteointegration to HIT, supporting their use to reduce surgical trauma in soft bone.

## Background:

Achieving predictable osseointegration is fundamental to the long-term success of endosseous implants. Primary stability, often assessed via insertion torque, is a critical determinant of early healing and implants survival [1]. In low-density (soft) bone, such as the posterior maxilla, achieving high insertion torque is challenging due to reduced bone mineral density and compromised mechanical properties [2]. Conventional protocols advocate for insertion torque values exceeding 35 Ncm to ensure stability, but excessive torque may induce bone compression, micro fractures and ischemic necrosis, potentially compromising healing [3]. Recent studies suggest that low insertion torque (LIT) strategies (typically 15-25 Ncm) may mitigate surgical trauma while still permitting secondary stability development through biological fixation [4]. Clinical investigations report favorable short-term outcomes with LIT in normal-density bone, with survival rates comparable to high-insertion-torque (HIT) implants [5]. However, the efficacy of LIT in soft bone remains controversial. Animal studies indicate that LIT implants exhibit reduced micromotion and enhanced vascularization during early healing, potentially favoring osteogenesis [6]. Conversely, some authors argue that insufficient initial torque risks implant failure in poor-quality bone due to inadequate mechanical interlocking [7]. A significant research gap persists regarding the comparative osteointegration of LIT and HIT implants, specifically in soft bone environments. Most existing literature focuses on immediate loading protocols or normal-density bone, with limited histomorphometric data from controlled models mimicking human soft bone [8]. Furthermore, the temporal dynamics of stability acquisition and bone remodeling around LIT implants are poorly understood. In addition to insertion torque, other biomechanical and biological factors influence osseointegration in soft bone. Thread design, implant surface modification and surgical site preparation all play significant roles in achieving stable bone-implant contact. Evidence suggests that under-preparation of the osteotomy can enhance mechanical engagement in low-density bone, but this approach risks excessive compression if combined with high torque values [9]. Conversely, LIT protocols combined with optimized implant macro- and microgeometry may allow for controlled engagement without compromising vascular supply, thereby fostering a more favorable biological environment for bone healing [10].

Furthermore, the dynamic process of osseointegration involves a critical balance between mechanical stability and biological adaptation. Early bone remodeling is driven by osteoclastic resorption followed by new bone apposition, processes that can be disrupted by excessive surgical trauma or micromotion beyond critical thresholds [1]. Implants placed with LIT may initially exhibit reduced primary stability but benefit from enhanced angiogenesis and early woven bone formation, potentially compensating for the lower torque values. The temporal pattern of stability acquisition-often described as a dip in mechanical stability during early healing-requires careful monitoring when adopting LIT strategies [2]. Clinically, the adoption of LIT in soft bone must be evaluated in the context of loading protocols. While immediate loading demands higher primary stability, delayed loading may permit the biological advantages of LIT to manifest without jeopardizing implant survival. Patient-specific variables such as bone density, systemic health and parafunctional habits also influence the choice of torque strategy. Therefore, rigorous experimental and clinical studies comparing LIT and HIT protocols in standardized soft bone conditions are needed to establish evidence-based guidelines for implant placement in challenging anatomical sites [3]. Therefore, it is of interest to evaluate the osteointegration of implants placed with low insertion torque versus conventional high insertion torque in a validated soft bone model. We hypothesized that LIT implants would achieve comparable histomorphometric and stability outcomes to HIT implants after 12 weeks of healing.

## Materials and Methods:

## Study design:

A randomized, controlled, split-mouth animal trial was conducted.

## Sample size:

Six adult female sheep (Ovis aries, age 2-3 years, weight 65-75 kg) were selected. A power analysis (α = 0.05, β = 0.2) based on prior bone-to-implant contact (BIC) data indicated that 10 implants per group were required to detect a 10% difference. Accounting for potential attrition, 12 implants per group (total 24) were used.

## Inclusion criteria:

[1] Healthy sheep with no metabolic bone disease.

[2] Femoral condyles with bone density < 500 Hounsfield units (HU) confirmed via preoperative CT.

## Exclusion criteria:

[1] Evidence of infection or prior surgery at implant sites.

[2] Systemic conditions affecting bone metabolism.

## Materials:

[1] Implants: 24 commercially pure titanium implants (4.1 mm diameter, 10 mm length, sandblasted, large-grit, acid-etched surface).

[2] Surgical Tools: Standardized drill kit (2.0-3.8 mm), torque wrench (range 5-50 Ncm), resonance frequency analysis (RFA) device (Osstell Mentor).

[3] Analysis Systems: Micro-CT (Skyscan 1272, Bruker), histology equipment (Exakt cutting/grinding system).

## Procedures:

[1] Preoperative Planning: Sheep underwent CT scanning. Femoral condyles were mapped for osteotomy sites, ensuring uniform bone density.

[2] Surgery: General anesthesia was induced. Bilateral femoral condyles were exposed via lateral approaches. Osteotomies were performed under copious saline irrigation using a standardized drilling protocol (1500 rpm, 50 Ncm). Implants were randomly assigned to LIT (15-25 Ncm) or HIT (35-45 Ncm) groups using a computer-generated sequence. Insertion torque was recorded. RFA was performed at placement and 12 weeks.

[3] Healing: Sheep were housed for 12 weeks with unrestricted mobility.

[4] Sample Harvesting: Euthanasia was performed via barbiturate overdose. Block sections containing implants were excised and fixed in 10% neutral buffered formalin.

## Analysis:

[1] Micro-CT: Scans were performed at 10 µm resolution. Bone volume fraction (BV/TV), trabecular thickness (Tb. Th) and bone-implant contact (BIC) were quantified using CTAn software.

[2] Histomorphometry: Specimens were dehydrated, embedded in methylmethacrylate and sectioned (200 µm). Sections were ground to 50 µm, stained with toluidine blue and analyzed under light microscopy. BIC was measured at 50x magnification.

[3] Stability: Implant stability quotient (ISQ) values were recorded via RFA.

## Statistical analysis:

Data were expressed as mean ± standard deviation (SD). Normality was assessed using Shapiro-Wilk tests. Independent t-tests compared LIT and HIT groups for continuous variables (BIC, BV/TV, Tb.Th, ISQ). Paired t-tests evaluated within-subject differences. Significance was set at p < 0.05 (SPSS v28.0).

## Results:

All implants remained osseointegrated at 12 weeks. Insertion torque differed significantly between groups (LIT: 22.4 ± 3.1 Ncm; HIT: 40.2 ± 3.8 Ncm; p < 0.001). Initial ISQ was lower in LIT implants (68.3 ± 2.5) than HIT (73.1 ± 2.8) (p < 0.001), but this difference resolved by 12 weeks (LIT: 72.5 ± 3.2; HIT: 74.1 ± 2.9; p = 0.15) (Table 1 - see PDF). Micro-CT analysis revealed no significant differences in BV/TV (LIT: 35.4% ± 5.1%; HIT: 38.2% ± 4.9%; p = 0.22) or Tb.Th (LIT: 0.21 ± 0.03 mm; HIT: 0.23 ± 0.04 mm; p = 0.11) (Table 2 - see PDF). Histomorphometry showed comparable BIC (LIT: 58.3% ± 7.2%; HIT: 62.1% ± 6.8%; p = 0.18). Bone remodelling was active in both groups, with osteoblasts lining implant surfaces and minimal fibrous encapsulation (Table 3 - see PDF).

## Discussion:

This study demonstrates that implants placed with low insertion torque (15-25 Ncm) achieve osteointegration comparable to conventional high-insertion-torque (35-45 Ncm) implants in soft bone after 12 weeks. Despite significantly lower initial stability, LIT implants exhibited equivalent bone-to-implant contact, bone volume fraction and final stability. These findings align with emerging evidence that moderate initial stability may suffice for successful osseointegration when biological healing is optimized [9]. The comparable BIC values (58.3% vs. 62.1%) and BV/TV (35.4% vs. 38.2%) between groups suggest that LIT does not compromise bone formation. This contrasts with earlier studies asserting that insertion torque < 30 Ncm increases failure risk in soft bone [10]. Our results support recent clinical data indicating that LIT implants achieve survival rates > 95% in low-density sites, provided micromotion is controlled during healing [11]. The absence of fibrous encapsulation in histology further confirms functional osteointegration. The resolution of initial ISQ differences by 12 weeks (72.5 vs. 74.1) highlights the role of secondary stability. LIT implants likely experienced reduced surgical trauma, preserving osteocyte viability and accelerating remodelling. This is consistent with animal studies showing that excessive torque induces bone microdamage, delaying healing [12]. The comparable trabecular thickness and osteocyte density further indicate similar bone quality around both implants. Our findings challenge the dogma that high insertion torque is mandatory for soft bone. Instead, they align with the "underpreparation" concept, where minimal osteotomy compression preserves bone biology [13]. However, the lack of significant differences may reflect the 12-week endpoint; longer studies could reveal divergence in remodelling patterns. Additionally, the sheep model, while validated for bone healing, may not fully replicate human oral conditions. In addition to the histomorphometric and stability outcomes, the biological plausibility of low insertion torque strategies lies in their capacity to preserve peri-implant vascularization. Excessive compression associated with high torque can compromise local blood supply, leading to delayed angiogenesis and impaired bone remodelling. In contrast, LIT protocols may maintain a more favourable microenvironment for osteoprogenitor cell recruitment and differentiation, thereby supporting balanced bone turnover [14]. This biological advantage could be particularly relevant in soft bone, where the intrinsic regenerative capacity is already limited by reduced trabecular density.

Moreover, the present findings support the paradigm shift toward individualized implant placement strategies based on patient-specific bone characteristics rather than rigid torque thresholds. Several recent clinical trials have emphasized that implant survival depends more on the biological response and loading conditions than on achieving a specific torque value [15]. Thus, clinicians should consider bone quality, implant design and surgical technique collectively, rather than relying solely on insertion torque as a determinant of long-term success. This integrative approach could enhance clinical decision-making in anatomically challenging regions such as the posterior maxilla. Finally, it is important to recognize the limitations of the present study. The 12-week observation period, while sufficient to demonstrate early osseointegration, does not provide insights into long-term remodelling dynamics or functional loading outcomes. Furthermore, although the sheep model offers a validated approximation of human bone physiology, interspecies differences in trabecular architecture and remodelling rates may affect extrapolation to clinical practice [16]. Future studies should therefore include extended follow-up durations, diverse animal models and randomized controlled human trials to validate the clinical applicability of LIT protocols [17, 18]. Future research should explore LIT in immediate-loading protocols and evaluate molecular markers of bone turnover. Clinical trials in human populations with type IV bone are warranted to confirm translational relevance.

## Conclusion:

We show that implants placed with low insertion torque (15-25 Ncm) achieve osteointegration comparable to high-insertion-torque (35-45 Ncm) implants in soft bone after 12 weeks. Despite lower initial stability, LIT implants exhibited equivalent bone-to-implant contact, bone volume fraction and final stability. Thus, LIT is a viable strategy to minimize surgical trauma in compromised bone, potentially expanding treatment options for patients with low-density bone.
